# Supplementary material for: QTL Mapping for Resistance to Bacterial Wilt Caused by Two Isolates of Ralstonia solanacearum in Chili Pepper (Capsicum annuum L.)
Source: Plants (Basel). 2022 Jun 10;11(12):1551. doi: 10.3390/plants11121551 (PMC9229654; doi:10.3390/plants11121551)
Supplement: Supplementary file 1 [file plants-11-01551-s001.zip › plants-1745232-supplementary.pdf]

Table S1. Summary of HRM markers developed in the ‘HS’ inoculated population.

Chromosome	Number of HRM markers developed	Number of HRM markers mapped
2	8	2
3	7	4
5	21	4
7	25	14
8	28	7
9	29	15
10	7	3
12	9	2
Total	134	51

Table S2. Summary of HRM markers developed in the 'HWA' inoculated population.

Chromosome	Number of HRM markers developed	Number of HRM markers mapped
2	2	1
3	2	2
5	30	6
7	20	9
8	4	3
9	17	4
10	2	2
12	2	4
Total	79	31

Table S3. Detailed information of HRM markers mapped in the 'HS' inoculated population.

Chr.	Marker name	Position (cM)	Forward primer	Reverse primer	SNP
2	C02_156631962-HRM	119.1	AGCTACGGCAGCATCTGTTT	CTACTGTTGCACCCACCTGA	G/T
	C02_156543304-HRM	119.2	GCTGAACCCGAGTCAAATTC	AAATTGCTCTTGACCAGTGGA	A/G
3	C03_248124064-HRM	250.3	AATGCTGACTTTAATGTGTTGTGA	AATAAACAAGACGCAACAACCATA	T/G
	C03_248135782-HRM	250.8	TCTTGCTACACAGGTAACGGATAA	TAAACCTTGTTGACCCATCTTTTT	G/A
	C03_247609503-HRM	251.6	GCTGCTACTTGGAACAACTGAAG	TCTCCTGTTGGACAGAATCTGATA	G/A
	C03_247556260-HRM	252.1	GCTATATTCTTAAGGTCCCTGAGGT	TTGGAAGTATTTGGCAATGTAAGA	C/T
5	C05_232032389-HRM	88.9	GCTGCTTGTCAAATTCTTTGATTT	AGGAAATAGGACCTACAACCACAA	A/G
	C05_232504724-HRM	91.2	GATACCTAATGATGGCTGTGTACG	GCAGCCTAACAGGTACCTAATGAT	T/C
	C05_232227275-HRM	91.7	TTTTGAGGCCAAATTAGTGTGTTA	TTTATCCAAAATCATGCCCTAACT	C/T
	C05_232869415-HRM	92.5	ACGTTCAATCCACAAGTACAAATG	TAGCGCTTCTGTTATTCCCATAGT	G/A
7	C07_221534108-HRM	131.2	GAAAAGGTTTATCGTTCAGGTGTT	AGGCAGTATACAGTGTCATTTCCA	T/G
	C07_221745500-HRM	131.6	AAACTGGGTGCAACATACTCAAC	CACCTCAGTTGAACTTGTCGTAGT	G/A
	C07_221715911-HRM	131.7	TAATACAGGCGCAAACACAGTT	TGCTAGGTTAGACCATACTGAACG	G/A
	C07_221772247-HRM	132.3	GCAAGTGATCAACCTGTCCTACT	CCAATGAATCTTTTTAAACCGAAA	C/T
	C07_222303778-HRM	132.9	ATAGCTGCTGCCAATATCCTCTAC	GATTAAAGAGCAGTTTAAGCGTGA	T/G
	C07_222343934-HRM	133.2	TGGTCCTACTTTCATATGTCGATG	ATGTTCCTTTGTTCTGAGCTTTTT	C/T
	C07_223207483-HRM	134.7	GACATGTCTACATGGGAGGTACTG	GTAGGCAAAACTCTCACAATTCAA	C/T
	C07_223975297-HRM	139.0	ACGATGGTCATCCTTCATGTTAC	ATCCCACTTGTGAGTATACATTGG	G/A
	C07_224234013-HRM	139.6	AAGCCAAAGTTGTTTCTTGTTTTT	TAGTCGTCGGCTACATGTATCTTC	T/C
	C07_224331024-HRM	140.1	TGATTTTTCTGCTCTTGTTTCATC	CACAAACAGGAGGTTCCTAAACTT	G/A
	C07_224452216-HRM	140.5	TGAACAAAACCAAGTGACATACAA	TGCTAAATATGTCAGCATCAGCTT	A/C
	C07_224429537-HRM	140.5	AGCTCTTGGCATCAAACTCTACTT	TCCTCCTATTCTATCTTTCGTTTCA	C/T
	C07_224926788-HRM	142.8	ATTTGAGAAGTTGAGTGAATAGGC	GCCAAGGCTGCAAAACTATATC	G/A
	C07_225015776-HRM	143.2	CGAAAGGAAACAATGCCTAATATG	GCTGCGACATTGTAGGTTGTTAT	C/T
8	C08_134064617-HRM	108.0	TCCGGAAGTATCCCCTTTTC	TCACCTGCTGCTTCCTTCTT	G/A
	C08_134003873-HRM	108.5	GTGTTGCCCTCCCCTATTTT	GGGAGCTGCACATTTCTTCT	C/T
	C08_136607972-HRM	111.7	CCTTCGTACATTCAAATCAACATC	ACCTTGTATTAGTCAATGCTGCAA	A/G
	C08_136569084-HRM	112.0	ATCTGTGATAGCAGCAGTCAAATC	ACCTGATCACTGGGATAAGAGAAG	C/T
	C08_136854234-HRM	112.5	TCAATACCCGTAATTCTTCTTTGTT	AGCAGCTTTTGCTTAGTCCCTAT	T/C
	C08_135774389-HRM	115.6	CAGTTCACATTAAGGTCATCCAGA	AAAGTTTCTTGGGGAAAAGAAATA	A/G
	C08_136034497-HRM	115.9	CACAAATAATTCTCATCGAAACGA	GAAGAACAGCTTGTTTTGATTGTC	T/G

Table S3. Continued.

Chr.	Marker name	Position (cM)	Forward primer	Reverse primer	SNP
9	C09_1812448-HRM	12.1	AAAATTCTAGCCATTTTCTTCGTG	CACAAATTTTCGGAGGAGGAG	A/G
	C09_2406987-HRM	12.3	CATTTCCTCCCCTCGTATCA	CAACAAGCTGCAGAAAAGCA	C/T
	C09_1831460-HRM	13.0	TGGTCAGAAATTCATAAGAACCAA	CTGTCGTTGAGTGGAACTACTGTT	A/G
	C09_2974163-HRM	13.5	ATGCGCTATGTCGGGTAAAC	CCTTTCCACCTGCACTTTTC	C/T
	C09_3486004-HRM	14.9	TTTCTCCCTGGCTTGTTCTG	AAGATGCAGTCACAGGCAAA	T/C
	C09_5494783-HRM	25.4	GGAAATTTTCAGGCTACATCAGTC	AGCCCGACCAATTTTTCAT	T/C
	C09_4050683-HRM	26.3	TGCCTGACGGATACAAAACA	TCGAGTGCACTTGTTGCTTC	C/T
	C09_5494729-HRM	28.3	ATGGGAAATTTTCAGGCTACATC	AGCCCGACCAATTTTTCATAA	A/G
	C09_5494635-HRM	28.3	AATTGACAAGGCTGCCAGAG	CCAAGGATTCACACTAGGCTACTC	G/A
	C09_5501529-HRM	28.3	TATAGCCTACGGTTTCCTAAGTGG	AGCCTGGTCAATTTTTATCCAA	C/T
	C09_6047262-HRM	29.8	TTATATCCAGTTTCTTGGACTTCG	ACGGAAGTTCTAAATGAGTTTTGG	A/G
	C09_6048971-HRM	29.8	CATGAAACATGGAAAGTTCTAAAG	CATATGGTTTTCTTGGTGCAG	G/A
	C09_6033720-HRM	30.3	ATGACCATTATCAGCATTGGAAC	CAAACGCTGCTTTTCTTTGAC	A/G
	C09_6493519-HRM	31.4	AGTTCATACAGTCTATGCATGGTG	TATTCTGACCTTAAACGTCGTTGC	G/T
	C09_7062729-HRM	34.1	AAAGATAAAATGATAGGCCACTCG	AAACTTATTTGCAAAACATGAGCA	A/G
10	C10_231720724-HRM	114.1	CCGACGTTAGGACAAAAATCA	CTTATCTGCACAGGGCTGCT	A/G
	C10_232102547-HRM	114.4	TTCCTTGGATCTGTGGGTTG	TGGCATCTAATCACGCCATA	A/G
	C10_232244800-HRM	115.2	CTTTCGCAGCCAATTTATGA	TCGCTAGAACAGGGAGAACAA	A/G
12	C12_10118193-HRM	50.3	TAGTTCATCTGCGTTAACTGCTTC	TTCTCATCGGAATATGGAACTACC	G/A
	C12_11060403-HRM	55.5	AAGTAGAATGAAAGCAGCCCACT	TGATCTTCTATCTTTTGTCCTACTCA	T/C

Table S4. Detailed information of HRM markers mapped in the 'HWA' inoculated population.

Chr.	Marker name	Position (cM)	Forward primer	Reverse primer	SNP
2	C02_156631986-HRM	113.2	AGCTACGGCAGCATCTGTTT	CTACTGTTGCACCCACCTGA	G/A
3	C03_248217246-HRM	216.8	CAAATGAAATGGCACATTATGCT	TATACCAACACATTCAATCCATCC	C/T
	C03_247557016-HRM	217.2	ATCTTGCGGCATTACAGAACTC	ATCCAATTCCAAAGGATACAAACA	G/A
5	C05_3865058-HRM	36.6	ATTCAGACAATAGCAGCATTAGCA	CTTAAGTCCCCTGAAGTTGATGAT	A/C
	C05_224016474-HRM	117.8	TTGTTAGTTTCATTGTCGCCTAAA	ATCCGGTTCTTGATAAGATCGAC	T/C
	C05_224018748-HRM	117.8	GCTTGAAACTCCATCAGATAGACA	TTACAGTAAGTTGTGACGGACCAG	G/A
	C05_232032712-HRM	155.1	GTAGACCCTTGTAGTCTGGTCCTT	TCATTCAGCTTTAGAGTAAAAGTGG	C/T
	C05_232869415-HRM	161.2	ACGTTCAATCCACAAGTACAAATG	TAGCGCTTCTGTTATTCCCATAGT	G/A
	C05_232188025-HRM	161.6	CGCAAAATTCTTGAGTTTCTTTTT	GGGTTGTGCCTAATAAAATCACAT	A/G
7	C07_115482180-HRM	75.8	TAACACCAGTGAAAAGTGCTCAAT	GATCATACATGTACAAAAATGGAGTG	A/G
	C07_78012800-HRM	75.9	TTTGTCAGAAAAAATGCATGTATGA	TGGAATGCTTAGGCATCAGATAAT	A/G
	C07_78012359-HRM	75.9	ATGAATAAAGGTCAGGGCTACATC	CTGTCAGTGGTATGCTTTGGTCTA	C/A
	C07_78011631-HRM	75.9	ATGACATAAAGAACATGAGCAAGC	AAATGTGGAACTCCAAAAATGAAT	A/G
	C07_115436147-HRM	76.0	TATGATCTTACCGAGTGATTCTGC	TATTAGTCCCCAATCAGCTATGGA	A/G
	C07_115436073-HRM	76.1	AGTTGATGCTGAAGTTGAAAAACC	AGACTCAAGTGGCAGAATCACTC	G/A
	C07_78012481-HRM	76.2	AGTTGGCGAATTTAATCCACTAGA	ATTGACCTTAACCTCTGCACTTTC	A/C
	C07_221718700-HRM	146.7	CCATTTTTGGGACAAATAGCAG	AAGTTCAAGATGAACCAAATCCAT	T/C
	C07_223209253-HRM	151.9	ACTAGAAACGTACCTTGCATTTCC	AGCTAAAATTCAGGTCTGTGGAAG	C/T
8	C08_134011994-HRM	80.2	CTGCAGCACGTGTAGTGGTT	CATGAAGATGCTCTGCCGTA	A/C
	C08_136569084-HRM	82.1	ATCTGTGATAGCAGCAGTCAAATC	ACCTGATCACTGGGATAAGAGAAG	C/T
	C08_136932188-HRM	83.1	CATTGATCTCAATTCCATCAAATC	CATACACATACTTCCGTTTCCAAG	G/A
9	C09_1814095-HRM	39.8	TGTCGGAAAATGATCAAAAAGTTA	AGCAGCTCAATAGCGAAACTAGAA	T/G
	C09_2406987-HRM	40.7	CATTTCCTCCCCTCGTATCA	CAACAAGCTGCAGAAAAGCA	C/T
	C09_5503170-HRM	63.6	GTGCTATAGCCCACGATTTCTAGT	CTCGGTCAATTTTACACCTGAAAC	T/C
	C09_6048824-HRM	66.0	AGTCTAGTATTGAACTCGCCAACA	GAAATAATTTTCAACTCCACAGCA	C/A
10	C10_232055780-HRM	174.2	CAAGTTCCTCTTCGGCTACG	CACCAGCATCAATGGAAAGA	C/A
	C10_231794580-HRM	174.3	TATTGCGGTGCAAATTGAAA	TTCTCTCGACACACACCAATG	T/C
12	C12_8116140-HRM	42.6	AGAATTGTCCGTCTCTTACCGTAG	TATGACTGGGCATGTTATTGTTGT	C/T
	C12_10117958-HRM	49.4	AGCTGCTACCAGAGCTATGATTTT	AACCAGCCTTTTCATAGTCTTCTG	C/T
	C12_210090697-HRM	115.5	TGACTGAGCATTTGTTCAGAAAAC	TATGAAGTTTATCTGCCGTTGATG	A/G
	C12_210087105-HRM	116.0	CTGCAGATTCCTGCAAGAAAAATA	ATAGTTGCATCTTTTGGAGCTCTT	C/T

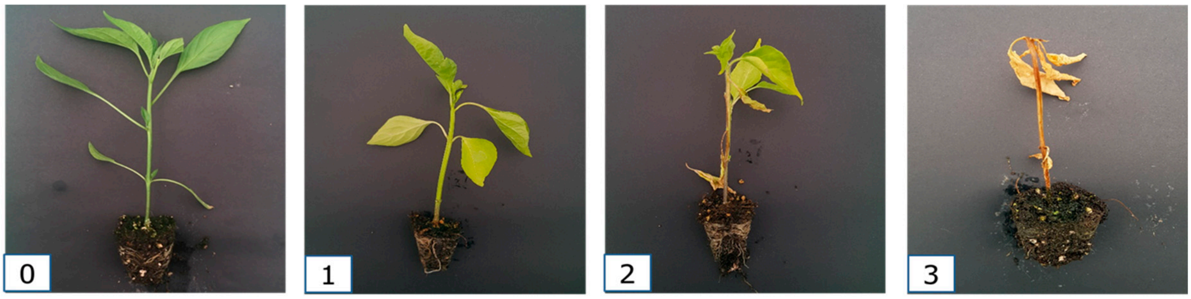

Figure S1. Symptoms of pepper seedlings caused by *Ralstonia solanacearum*. 0, no symptoms; 1, cotyledon etiolated; 2, most of leaves and stem wilted; 3, whole plant wilted or dead.

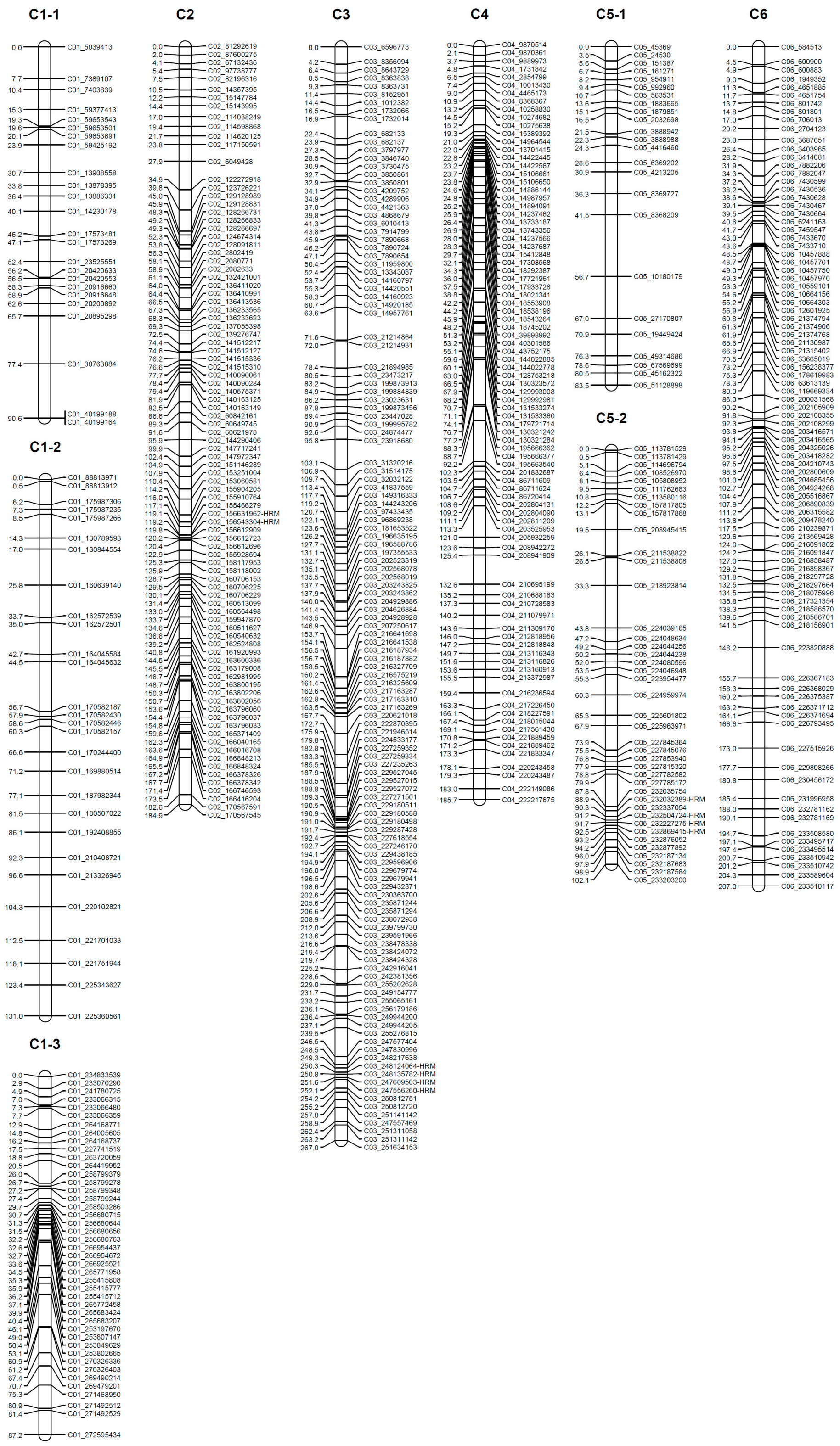

Figure S2. The pepper genetic linkage map consisting of 1,168 SNPs derived from GBS analysis constructed in the 'HS' inoculated F_2 population from a strongly resistant cultivar 'Konesian Hot'. Bar left number, genetic position (cM); bar right name, name of SNP marker.

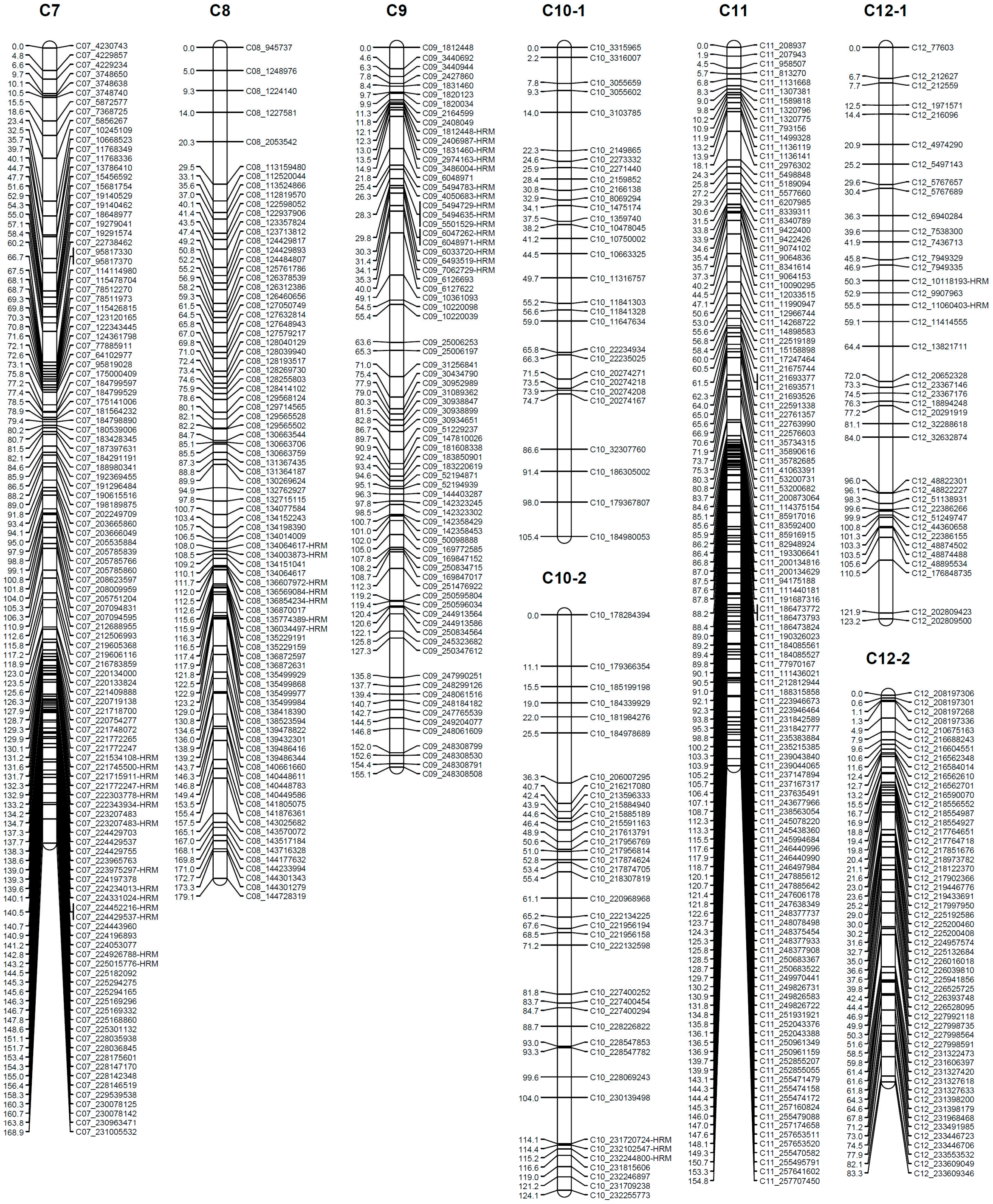

Figure S2. Continued.

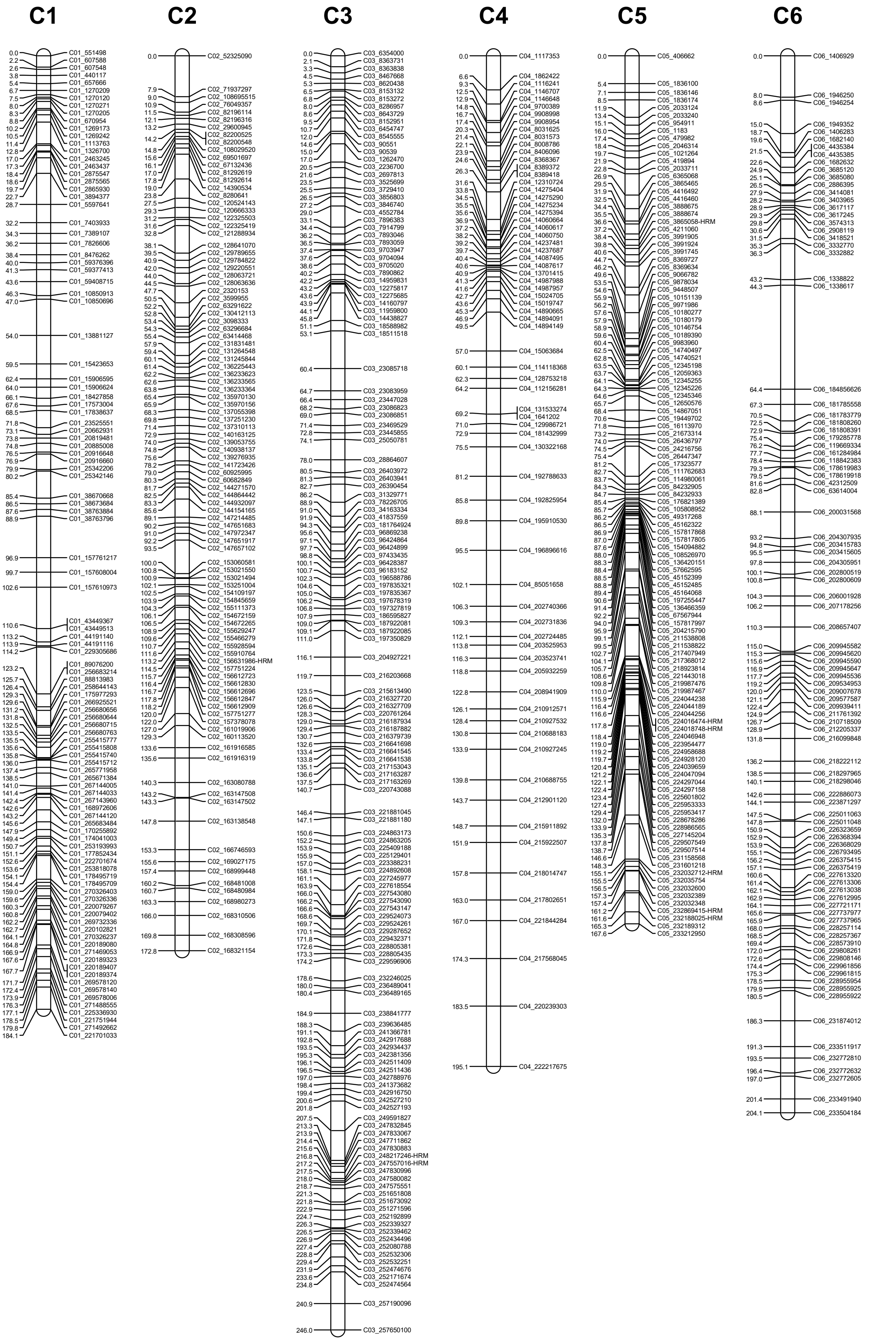

Figure S3. The pepper genetic linkage map consisting of 1,317 SNPs derived from GBS analysis constructed in the ‘HWA’ inoculated F_2 population from a strongly resistant cultivar ‘Konesian Hot’. Bar left number, genetic position (cM); bar right name, name of SNP marker.

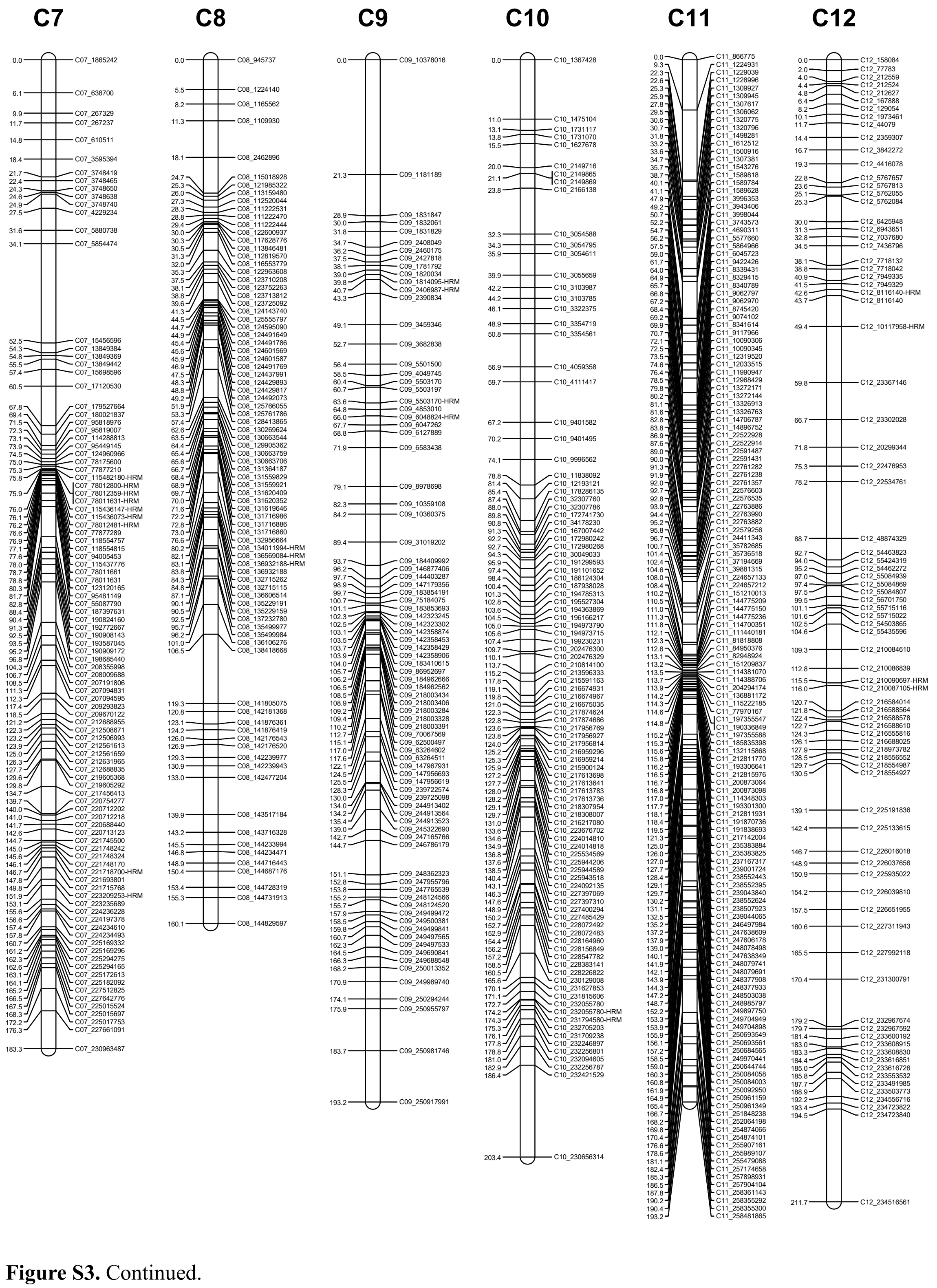

Figure S3. Continued.

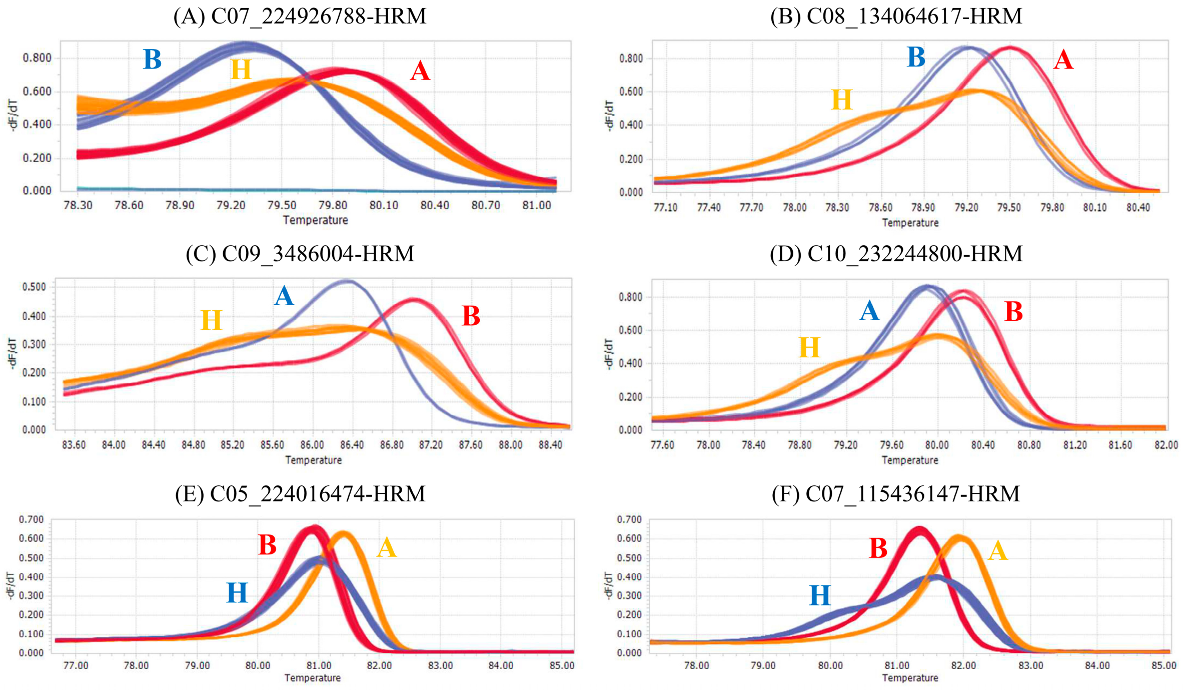

Figure S4. Melting curves of HRM markers linked to QTLs for bacterial wilt resistance in *Capsicum annuum*. A, maternal homozygous genotype; B, paternal homozygous genotype; H, heterozygous genotype. (A) C07_224926788-HRM was linked to the QTL *Bwr6w-7.2* conferring resistance to 'HS' isolate of *Ralstonia solanacearum*. (B) C08_134064617-HRM was linked to the QTL *Bwr6w-8.1* conferring resistance to 'HS' isolate of *R. solanacearum*. (C) C09_3486004-HRM was linked to the QTL *Bwr6w-9.2* conferring resistance to 'HS' isolate of *R. solanacearum*. (D) C10_232244800-HRM was linked to the QTL *Bwr6w-10.1* conferring resistance to 'HS' isolate of *R. solanacearum*. (E) C05_224016474-HRM was linked to the QTL *Bwr6w-5.1* conferring resistance to 'HWA' isolate of *R. solanacearum*. (F) C07_115436147-HRM was linked to the QTL *Bwr6w-7.1* conferring resistance to 'HWA' isolate of *R. solanacearum*.
